# Insights Into Bone Marrow Niche Stability: An Adhesion and Metabolism Route

**DOI:** 10.3389/fcell.2021.798604

**Published:** 2022-01-18

**Authors:** Driti Ashok, Laura Polcik, Svenja Dannewitz Prosseda, Tanja Nicole Hartmann

**Affiliations:** ^1^ Department of Internal Medicine I, Faculty of Medicine and Medical Center, University of Freiburg, Freiburg, Germany; ^2^ University of Freiburg, Faculty of Biology, Freiburg, Germany

**Keywords:** Bone marrow, microenvironment, adhesion, metabolism, integrin, VLA-4, α4β1, AML

## Abstract

The bone marrow microenvironment provides critical cues for hematopoietic stem cell (HSC) self-renewal and differentiation and contributes to their malignant conversion. The microenvironment comprises a complex mixture of multiple cell types, soluble factors, and extracellular matrix in specialized regions termed ‘niches.’ Positioning of the various cellular players within these niches depends on their repertoire of adhesion molecules and chemotactic signaling, involving integrins and chemokine receptors and the corresponding intracellular players such as kinases and GTPases. The mechanical role of adhesion is to control the strength and morphology of the cell-cell and cell-extracellular matrix contacts and thereby the energy needed for the optimal localization of cells to their surroundings. While it is clear that biomechanical adhesive bonds are energetically expensive, the crosstalk between cell adhesion and metabolic pathways in the normal and malignant microenvironment is far from understood. The metabolic profile of the various cell types within the niche includes key molecules such as AMPK, glucose, mTOR, and HIF-1α. Here, we describe our most recent understanding of how the interplay between adhesion and these metabolic components is indispensable for bone marrow niche stability. In parallel, we compare the altered crosstalk of different cell types within the bone marrow niches in hematological malignancies and propose potential therapeutic associations.

## Introduction

Hematopoiesis is a homeostatic process that starts with hematopoietic stem cells (HSCs) and ultimately leads to the formation of mature blood cells of the myeloid and lymphoid lineages. This process is supported by various cell types and soluble factors within the bone marrow (BM) microenvironment. Hematopoietic malignancies arise when the balance within the BM is disturbed, e.g., by DNA damage causing genetically abnormal hematopoietic cells (Hanahan and Weinberg, 2011; Bakker and Passegué, 2013) and converting HSCs into their malignant counterparts, the so-called leukemia stem cells (LSCs) or leukemia-initiating cells (LICs). These malignant cells further alter their microenvironment at the expense of normal hematopoiesis to facilitate their own growth and neoplastic processes (Wang and Zhong, 2018).

Retention within the BM is required to allow survival, proliferation, and differentiation of HSCs. For this purpose, HSCs express adhesion molecules such as integrins, selectins, and CD44 that bind their specific ligands presented by other cells, e.g., stromal cells (Klamer and Voermans, 2014; De Grandis et al., 2016; Levesque and Winkler, 2016; Mitroulis et al., 2020). Some of these molecules regulate HSC maintenance and retention and serve as biomarkers to determine the extent of malignant transformation (Kulkarni and Kale, 2020), e.g., the integrin very late antigen-4 (VLA-4, α4β1, see later chapters).

Normal hematopoiesis and malignant transformation are shaped by bioenergetics, biosynthesis, and the redox balance (Boroughs and Deberardinis, 2015; Deberardinis and Chandel, 2016; Pavlova and Thompson, 2016). Malignancies are often accompanied by hypoxia and altered ATP levels, which reflect the increased nutrient demand that is characteristic of malignant cells (Kim et al., 2006; Hardie, 2008). Adhesive processes are energetically expensive and thus modulated by bioenergetics. However, the crosstalk between cell adhesion and metabolic pathways in the normal and malignant microenvironment is far from understood.

This review describes how various hematopoietic and non-hematopoietic cell types interact within the normal and malignant microenvironment. We delineate the various factors that modulate integrin-dependent adhesion within the BM and the consequences of an altered adhesion-metabolism interplay for BM microenvironment stability.

## Bone Marrow Compartmentalization: Cellular Structure and Adhesive Factors

**FIGURE 1:**
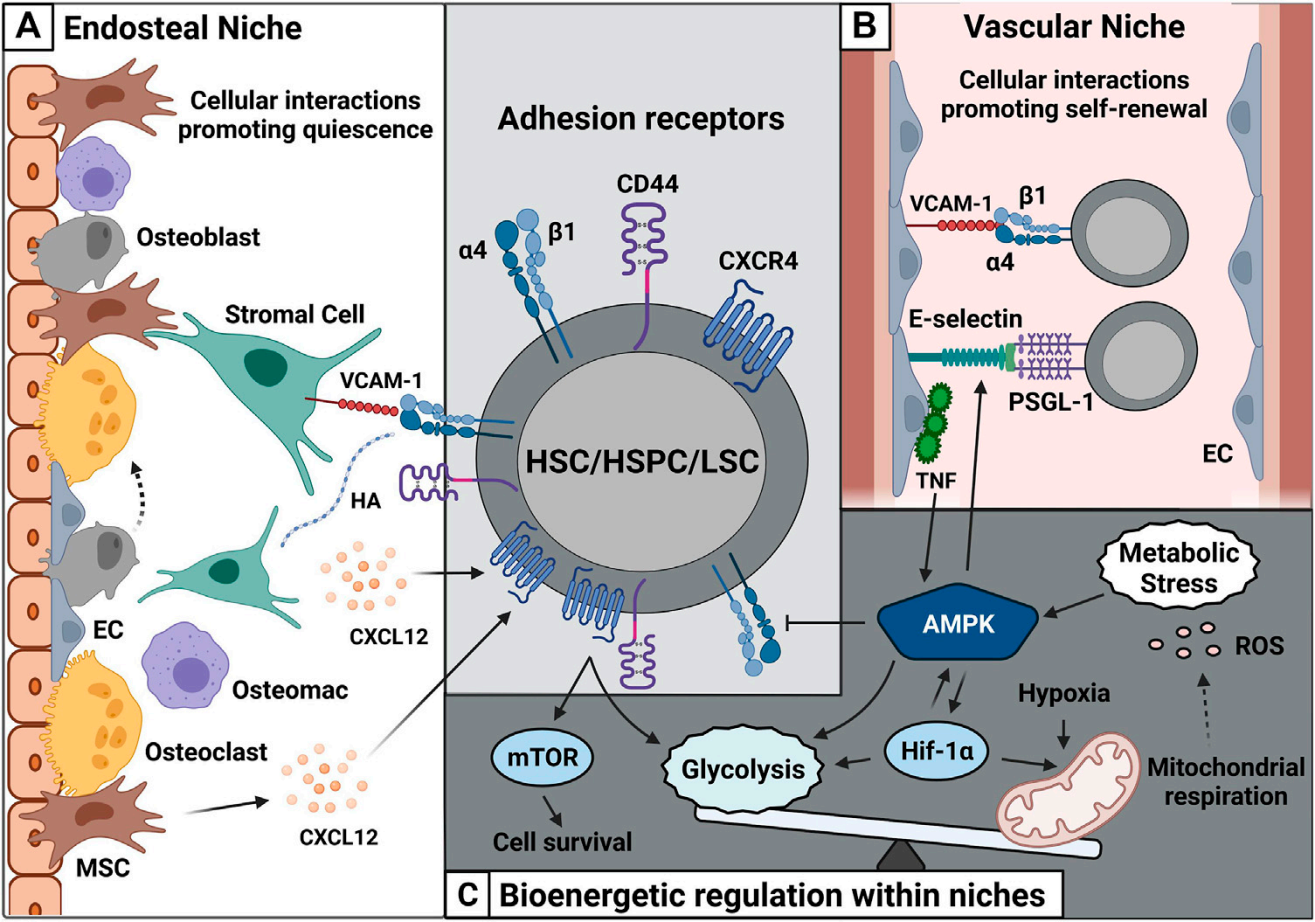
Cell-cell interactions within the BM microenvironment are shaped by adhesion and metabolism. **(A)** HSC/HSPC/LSC express adhesion receptors such as α4β1, CD44, and CXCR4. These receptors interact with their specific ligands such as VCAM-1, HA, and CXCL12 on non-hematopoietic cells such as stromal cells, MSCs and osteoblasts and contribute HSC quiescence. **(B)** The vascular niche made up of sinusoids contains numerous ECs. These ECs express large amounts of E-selectin and VCAM-1 and promote HSC/HSPC/LSC self-renewal. **(C)** Enhanced CXCR4-CD44 cooperativity can promote leukemic cell survival *via* mTOR and elevates glycolytic influx. Metabolic stress further increases AMPK activity promoting glycolysis and Hif-1α expression. High amounts of Hif-1α create a hypoxic environment followed by release of ROS. Abbreviations: HSC/HSPC, hematopoietic stem cell/hematopoietic stem and progenitor cell; LSC, leukemic stem cell; MSC, mesenchymal stem cell; EC, endothelial cell; VCAM-1, vascular cell-adhesion molecule-1; HA, hyaluronic acid; CXCL12, C-X-C Motif Chemokine Ligand 12; PSGL-1, P-selectin glycoprotein ligand-1; CXCR4, CXC receptor 4; mTOR, mammalian target of rapamycin complex; AMPK, adenosine-5′-monophosphate-activated protein kinase; Hif-1α, hypoxia-inducible factor α; ROS, reactive oxygen species.

Two types of niches exist in the BM, namely, the endosteal and the vascular niche. The endosteal niche promotes HSC (Lin^−^ CD34^+^ CD38^−^) quiescence due to its unique composition (Nilsson et al., 1997; Wilson et al., 2007; Casanova-Acebes et al., 2013). It is composed of spongeous bone and specialized cells such as osteoblasts and osteoclasts (Figure 1A) (Nilsson et al., 1997; Wilson et al., 2007) and is enriched with a network of arterioles and sinusoids. Hematopoietic stem and progenitor cells (HSPCs) surround the surface of the endosteum. Along the sinusoidal belt, endothelial cells and mesenchymal progenitors are primed to give rise to osteoblasts (Nombela-Arrieta et al., 2013). Osteoblasts are important players within the endosteal niche (Xie et al., 2009), although osteoblast depletion in transgenic mice did not affect HSC numbers (Visnjic et al., 2001; Bowers et al., 2015; Yu et al., 2015), yet altered their cycling capacity (Bowers et al., 2015; Yu et al., 2015). Additionally, osteoblast depletion reduced pre-pro- and pro-B cell numbers in the BM (Zhu et al., 2007; Yu et al., 2015).

Furthermore, HSC survival depends on the interaction with the vascular network, comprising arteries that enter the BM and subdivide into numerous arterioles. The arterioles traverse the BM along with specialized capillaries, the so-called sinusoids (Figure 1B) (Kopp et al., 2005). These vessels lack a continuous basal lamina, and their open pores facilitate the passing of white blood cells from the BM while promoting hematopoietic cell proliferation and differentiation. The term ‘vascular niche’ describes this unique environment. Its self-renewing HSC pool is closely associated with BM endothelial cells along sinusoidal vessels towards the center of the BM (Figure 1B). This was confirmed by the knockout of the glycoprotein 130 (gp130) receptor in ECs, leading to reduced HSC numbers (Yao et al., 2005). The survival of ECs from Tie-2 gfp mice was co-dependent on HSCs *in vitro,* even in the presence of growth factors (Li et al., 2004).

## Adhesive Factors and Metabolic Components in Leukemia

The interaction of HSC with their niche requires communication *via* cell surface receptors, that mediate their adhesion to this environment. Particularly CD44, CXCR4 and the integrin α4β1 (VLA-4) play important roles in regulating HSPC trafficking, self-renewal, proliferation, and differentiation. These molecules extensively interact with each other and influence each other’s function, with the integrin α4β1 being activated by CD44 and CXCR4. The CD44 family comprises different variants of glycoproteins with extensive posttranslational modifications. This regulates the binding of CD44 to its ligands and promotes heterodimerization with other surface molecules, such as α4β1, thereby modulating integrin function (Yu et al., 2021; (Gutjahr et al., 2021). Chemokines such as the CXCR4 ligand CXCL12 also serve as integrin activators. Binding of CXCL12 to CXCR4 activates a rapid cascade of intracellular signaling events that eventually direct the integrin conformation to a high affinity state. This high affinity state allows a strong adhesion of the cells to the substrate.

Once the crosstalk of these three receptors is altered, leukemogenesis is driven. For example, CD44 cooperativity with α4β1 triggers adhesion of leukemic cells to stromal cells (Gutjahr et al., 2021), inducing prosurvival signaling pathways. Similarly, CD44 interacts with CXCR4, which can promote chemoresistance in AML (Yu et al., 2021).

CXCL12 is secreted by all stromal cells in the BM. CXCL12 abundant reticular (CAR) cells/Leptin receptor-positive cells (LepR^+^) (Seike et al., 2021) are a subset of VCAM-1^+^ reticular cells that promote HSC activity (Omatsu et al., 2010). Cell-specific depletion of CXCL12 diminished CAR cells, followed by a marked decrease in HSC proliferation (Omatsu et al., 2010).

Furthermore, CXCL12 knockout in endothelial cells leads to modest loss of self-renewing activity (Greenbaum et al., 2013). Similarly, the co-culture of ECs with HSCs provided a growth advantage, increasing HSC numbers (Butler et al., 2010). In leukemic conditions, such as T-cell acute lymphoblastic leukemia, CXCL12 knockout in ECs suppressed tumor development, indicating a dependency of the tumor on the vascular environment (Pitt et al., 2015). In addition, CXCL12 expression on osteoblasts is decisive for BM lymphoid progenitor populations and their reconstitution capacity (Greenbaum et al., 2013); Zhu et al., 2007). Furthermore, differentiated osteoblasts regulate osteoclast differentiation and function *via* cytokines such as macrophage-colony stimulating factor (M-CSF), osteoprotegerin (OPG), and OPG ligand (OPGL) (Yasuda et al., 1998). Osteoclasts are F4/80 low or negative macrophages that mediate bone resorption at the endosteal surface (Figure 1A) (Lacey et al., 1998). Additionally, the endosteum contains F4/80 (high) expressing macrophages called osteomacs that contribute to bone remodeling (Chang et al., 2008). Osteomacs surround osteoblasts (Chang et al., 2008; Winkler et al., 2010), CXCL12-abundant reticular cells (Casanova-Acebes et al., 2013), and Nestin^+^ mesenchymal stem cells (MSCs). Osteomacs and Nestin^+^ MSCs synergize in maintaining a high-adhesive niche. Upon osteomac depletion, Nestin^+^ cells display reduced levels of the adhesive components CXCL12, VCAM-1, and Angpt-1 (Chow et al., 2011), which are essential for HSC retention within the BM. In malignancy, Nestin^+^ MSCs may facilitate this crosstalk as they are known to support leukemogenesis.

**FIGURE 2:**
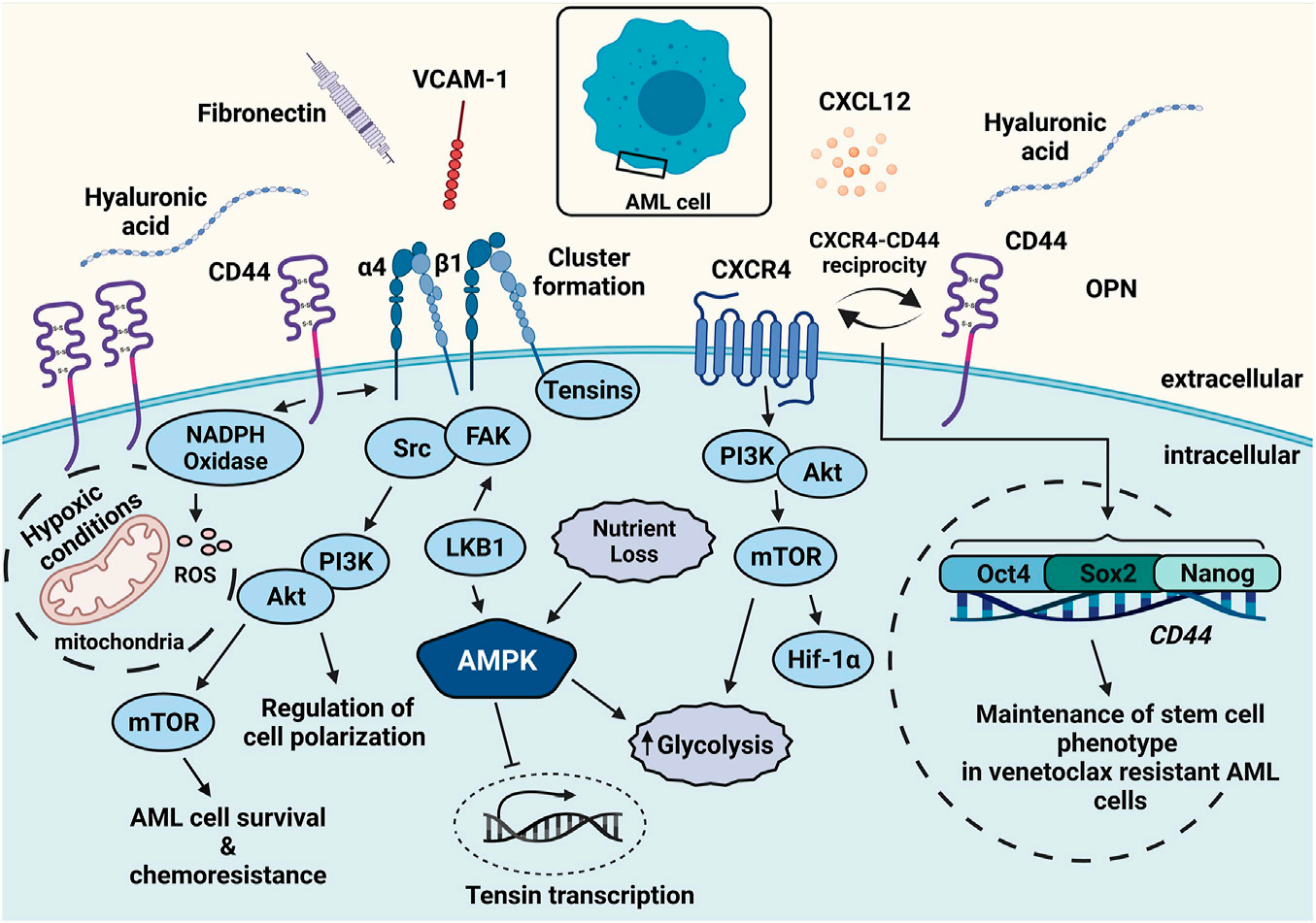
Leukemic cell survival can be augmented by adhesion and metabolic components. AML cells express high amounts of CD44, CXCR4, and α4β1. Ligands such as HA, CXCL12, and VCAM-1 secreted by stromal cells activate the receptors and render chemoresistance. CD44-mediated activation and clustering of α4β1 leads to adhesive hubs and promotes mTOR activity. CXCL12 interaction with CXCR4 can be followed by enhanced glycolysis. CXCR4 interaction with CD44 confers a cancer stem cell phenotype *via* Oct4, Sox2, and Nanog expression. Elevated CD44 expression increases ROS levels *via* NADPH oxidase and further promotes malignancy. Abbreviations: FAK, focal adhesion kinase.

CXCL12 enhances the survival capacity of AML cells and supports chemoresistance (Yu et al., 2021). It also induces phosphorylation of mTOR (Braun et al., 2016), which has been linked to increased glycolysis and translation of HIF-1α (Figure 2) (Konopleva et al., 2009). The siRNA-mediated knockdown of CXCL12 in AML cell lines and primary AML cells reduces glucose levels and mTOR expression (Braun et al., 2016). This phenomenon was recapitulated using the CXC receptor 4 (CXCR4) inhibitor plerixafor (AMD3100) (Uy et al., 2012). Notably, CXCR4 cooperates with the glycoprotein CD44 in AML, thereby enhancing resistance to the BCL-2 inhibitor venetoclax (Yu et al., 2021). This cooperation conferred a phenotype attributed to cancer stem cells (Cd44^high^, CXCR4^high^) (Yu et al., 2021) followed by high expression of embryonic stem cell core transcription factors such as Sox2, Oct4, and Nanog (Figure 2).

In fact, CD44 is one of the first-described cancer stem cell markers and key homing receptors (Naor et al., 2002; Jin et al., 2006). Alternative splicing of the *Cd44* gene gives rise to numerous variants (CD44v) (Naor et al., 2002; Jin et al., 2006). Normal cells expressing the standard isoform (CD44s) usually require activation and the induction of CD44 isoforms to bind their respective ligands, particularly hyaluronic acid (HA) and osteopontin. In contrast, tumor cells constitutively express CD44v and can bind ligands without further stimulation (Naor et al., 2002).

CD44 variants v6, v8, v9, and v10 (numbering based on expressed exons) are found in normal peripheral blood mononuclear cells (Dougherty et al., 1991; Bendall et al., 2000), yet at a low abundance. In hematological malignancies, these variants are primarily associated with non-Hodgkin’s lymphoma, myeloma, and leukemia, while in solid tumors, they are associated with breast and colon cancer (Legras et al., 1998; Asosingh et al., 2000; Medina et al., 2010; Guo and Frenette, 2014). Multifaceted properties of CD44 have been deciphered as links between LSCs and their BM microenvironment (Jin et al., 2006). One of these many links is found in the metabolic shift in the microenvironment that involves elevated production of reactive oxygen species (ROS) due to oncogene activity (Figure 2), along with an increase in ROS-driving enzymes such as NADPH oxidase. CD44v8-10 elevates the synthesis of reduced state glutathione (GSH) *via* its cysteine-glutamate antiporter system (xcT) (Ishimoto et al., 2011). This phenomenon aids cancer cells in overcoming elevated ROS levels, e.g., in the context of aerobic glycolysis (Warburg effect) (Ishimoto et al., 2011). Blocking CD44 by monoclonal antibodies leads to the terminal differentiation of leukemic cells, thereby reducing their potential for engraftment (Jin et al., 2006). The role of CD44 in maintaining the stemness character of leukemic cells can be attributed to either its direct binding to ligands or to its cooperation with other surface receptors, which eventually shapes the niche (Goodison et al., 1999; Orian-Rousseau and Sleeman, 2014; Gutjahr et al., 2021; Yu et al., 2021). For example, HA binding to CD44 on malignant cells activates the integrin α4β1, enhancing strong adhesion to VCAM-1, FN, and laminin (Grassinger et al., 2009; Gutjahr et al., 2021). Strong adhesion is mechanically based on α4β1 clustering upon the CD44-α4β1 cooperation and promotes stromal cell-leukemia cell contacts that act as metabolically-relevant “adhesive hubs” (Figure 2).

The cooperation of CD44 with α4β1 is one example of the so-called inside-out signaling of integrins, which serves as a means to integrate extracellular cues in cellular responses. Several possible signaling pathways and processes are involved in inside-out signaling, which starts with the activation of cell surface receptors, e.g., cytokine-, chemokine-, or antigen receptors. The activated surface receptors then induce an intracellular signaling cascade involving phosphoinositide 3-kinase (PI3K) and PLC-γ, among others, which eventually culminates in a high-affinity integrin status (Harzschel et al., 2020). The PI3K/Akt signaling pathway is also relevant for stromal cell-mediated chemoprotection of leukemic cells and for α4β1 downstream signaling to metabolic pathways *via* the master regulator (mTOR) (Shishido et al., 2014; Guerra and Issinger, 2020; Harzschel et al., 2020). Focal adhesion kinase (FAK) interaction with integrins has proven essential for the metabolic regulation of tumor cells (Yin, 2011), leading to excessive glucose consumption *via* the PI3K/Akt pathway (Bisht and Dey, 2008; Paik et al., 2009). In AML, three-dimensional access to VCAM-1 using coated beads triggers high phosphorylation levels of Akt, ERK, and mTOR (Gutjahr et al., 2021), and this downstream signaling is intensified by the association of Src and FAK (Zhou et al., 2016; Gutjahr et al., 2021). FAK activation by integrins also triggers the insulin growth factor receptor-1 resulting in sustained PI3K activation (Andersson et al., 2009). This particularly facilitated the proliferation of leukemic cells. In solid tumors, reciprocal interaction between β1-integrins and glucose metabolism has also been reported. In breast cancer cells, glucose metabolism is promoted by β1-integrin signaling through Twist, a regulator of epithelial-mesenchymal transition (EMT) (Yang et al., 2016), while knockdown of Glut1 in breast cancer cells lowers integrin β1 levels followed by reduced Src and FAK expression (Oh et al., 2017). With metabolic deregulation being a hallmark of both solid and hematological malignancies, the involvement of integrins and the underlying mechanisms of cell adhesion changes warrant further investigation.

## Hypoxia

Hypoxia regulates cell proliferation and survival *via* hypoxia-inducible factor 1-alpha (HIF-1α), mammalian target of rapamycin complex (mTORC), and adenosine-5′monophosphate-activated protein kinase (AMPK) (Kim et al., 2006; Hardie et al., 2012; Dibble and Manning, 2013).

The endosteal niche is widely believed to be hypoxic and shows oxygen levels of less than 2% (Spencer et al., 2014). This has been attributed to the quiescence of HSPCs that reside within the niche (Spencer et al., 2014). A report by Arrieta and others (Nombela-Arrieta et al., 2013) contradicted this traditional viewpoint. Using quantitative 3D imaging, the authors found that the hypoxic profile of HSPCs is independent of their cell cycle state and anatomical positions within the BM, implying that the tendency towards hypoxia could be driven by the cell populations within the BM rather than their location.

HIF-1α is one of the many critical factors driving glycolysis and mitochondrial respiration within the BM, with higher levels in hypoxic conditions (Figure 1B) (Papandreou et al., 2006). HSC survival is maintained by high rates of glycolysis (Simsek et al., 2010). For example, in quiescent HSCs, HIF-1α minimizes ROS generation and favors pyruvate dehydrogenase kinase expression (Takubo et al., 2013). When *HIF-1α* is knocked out in HSCs, glycolysis rates drop, and mitochondrial metabolism levels are elevated. This prevents pyruvate from entering the tricarboxylic acid cycle, thereby preventing mitochondrial respiration.

Integrin function is modulated by different oxygen levels in a partially HIF-dependent manner. For example, integrin α5β1-dependent upregulation of mast cell adhesion to fibronectin was observed in oxygen tensions as low as 1%. This upregulation was accompanied by increased Akt phosphorylation and sensitivity towards PI3K inhibition, indicating a hypoxia-driven integrin activation *via* the PI3K signaling pathway (Pastwinska et al., 2020).

## AMPK

Glycolysis is influenced by AMPK (Almeida et al., 2004), which allows cancer cells to survive metabolic stress (Figure 1C) (Jiang and Nakada, 2016). This energy sensor and central regulator for cellular metabolism plays a crucial role in growth and metabolic programming and is activated during cellular stress such as nutrient deficiency. Notably, AMPK is the most prominent factor known to link integrins and metabolism. The metabolic signaling *via* AMPK extensively influences adhesion mechanisms, e.g., the AMPK-activating kinase LKB1 regulates focal adhesion kinase (FAK), thereby controlling cell migration and cell polarization *via* PI3K localization (Mihaylova and Shaw, 2011). LKB1 expression is also necessary to maintain HSC quiescence. *Lkb1* abrogation resulted in enhanced cycling of HSCs and subsequent depletion (Nakada et al., 2010). AMPK phosphorylation of HSCs also required Lkb1.

AMPK-guided cell adhesion is regulated *via* the tensin family of proteins. Tensins bind β1-integrins and support their activation (Georgiadou et al., 2017) with the integrin adaptor talin. AMPK downregulates tensins and thereby negatively regulates β1-integrin activity. Factors that arrest gluconeogenesis, such as loss of nutrients, enhance AMPK activity. We propose that this results in reduced integrin-mediated adhesion in malignancies. However, AMPK levels have to be tightly balanced and thus both, enhanced and suppressed, AMPK activity can hinder cell migration (Nakano et al., 2010; Yan et al., 2015). This is molecularly based on dysregulation of the cytoskeletal arrangements during migration, with Rac activity at the front of the migrating cell being in dynamic balance with Rho A activity at the rear of the cell. Augmented AMPK phosphorylates its substrate, Pdlim5, higher phosphorylation levels of which down-regulates Rac1 expression at the leading edge of the cell (Yan et al., 2015). In contrast, suppressed AMPK activity alters microtubule polymerization (Nakano et al., 2010). While actomyosin filaments and the ECM mechanically regulate cell migration, the dual role of AMPK provides a possible explanation for the observed discrepancies in AMPK-mediated effects on integrin activity.

A reciprocal response from integrins is observed in regulating metabolic signaling. For instance, integrin-linked kinase (ILK) regulates factors upstream of YAP/TAZ, thereby promoting YAP/TAZ activity within the nucleus. This might be assisted by the TEAD family of transcription factors, which directly interact with AMPK (Ata and Antonescu, 2017). Indeed, the release of cyclic AMP, an activator of AMPK (Omar et al., 2009) supports the survival of B-lineage acute lymphoblastic leukemia cells. This suggests that regulating the cAMP pathway could serve as a therapeutic target in leukemia (Perez et al., 2016).

Selectins, CD44 and integrins strengthen the interaction between ECs and HSCs. During inflammatory conditions, selectins aid leukocytes in binding to endothelial cells and establish leukocyte rolling on the vasculature (Winkler et al., 2012). Constitutive E-selectin expression on ECs guides HSC activity (Schweitzer et al., 1996). Characterization of E-selectin knockout mice (Sele^−/−^) revealed slower HSC division, suggesting enhanced HSC quiescence and self-renewal capacity (Winkler et al., 2012). In BCR-ABL1-induced chronic myelogenous leukemia (CML)-like myeloproliferative neoplasia (MPN), Sele^−/−^ mice showed lower engraftment rates of leukemia compared to the wild-type, suggesting a homing defect (Krause et al., 2014). Endothelial damage occurs during malignancy and *via* irradiation and chemotherapy (Lyu et al., 2019; Wijerathne et al., 2021). As a result, this dislodges the vascular endothelium, creating impaired vascular permeability and elevating vascular fibrosis. In TNF-stimulated ECs, overexpression of dominant-negative AMPK (AMPKα2^−/−^) resulted in higher levels of NF-ĸB and E-selectin. AMPK activation in endothelial cells by vasculoprotective agents, such as metformin and VEGF, triggers several biological effects beneficial to vascular homeostasis (Cheng et al., 2007). These involve suppression of hyperglycemia-induced generation of ROS (Kukidome et al., 2006), alleviation of free fatty acid-induced lipotoxicity (Mccarty, 2005), and inhibition of nuclear factor-κB activation by tumor necrosis factor-α (Hattori et al., 2006). AMPK activity triggers endothelial progenitor cell differentiation *in vitro* upon VEGF stimulation (Li et al., 2008). Highly phosphorylated AMPK was associated with high levels of VE-cadherin and the integrin ligand ICAM-1, while AMPK inhibition reduced expression of these markers, suggesting the involvement of AMPK in endothelial progenitor cell differentiation (Li et al., 2008). In AML, a dense vascular compartment is a hallmark of poor prognosis (Shih et al., 2009) and BMECs are known to support AML cell quiescence (Ding et al., 2012; Ding and Morrison, 2013). Treatment with the tubulin polymerization inhibitor combrestatin altered the shape of BMECs, depleting VCAM-1 and VE-cadherin in AML cells, leading to increased peripheral blood cycling of AML cells. Combrestatins trigger ROS production in AML cells, and in combination with cytarabine, decrease leukemic cells within the murine BM (Bosse et al., 2016).

## Therapies Targeting the BM Microenvironment

Leukemia is marked by genetic and epigenetic alterations in hematopoietic cells and their respective progenitors (De Beauchamp et al., 2021). Myeloid leukemia (CML and AML) is particularly faced with the challenge of targeting cells that are treatment-resistant due to the large repertoire of mutations that arise, namely, point-mutations associated with BCR-ABL in CML or FLT3-TKD in AML (Corbin et al., 2011; Hamilton et al., 2012). Though widely implemented in clinical trials, tyrosine kinase inhibitors are not sufficient to eradicate minimal residual disease as they preferentially target differentiated cells (Corbin et al., 2011; Hamilton et al., 2012). An alternate route to target these malignant cells might be to interfere with metabolism. Preclinical data in CML showed that CD34^+^ cells display an oxidative phenotype marked by low levels of fatty acids such as oleic and linoleic acids and an increase in TCA metabolites, namely, glutamate and aspartate (Kuntz et al., 2017). Administration of the antibiotic tigecycline blocked levels of aspartate and, upon combination with the kinase inhibitor imatinib, arrested colony formation (Kuntz et al., 2017). Similarly, in CD44-overexpressing CD44^high^ K562 cells, non-steroid anti-inflammatory drugs (NSAIDs) administration triggered autophagy followed by increased sensitivity to heat shock protein inhibitor 17-AAG (Moon et al., 2019). This was supported by the activation of AMPK, and thus, inhibition of Akt/mTOR/p70S6K/4EBP1 activity. Autophagic cell death eventually led to the downregulation of CD44, Oct4, and c-Myc in these cells (Moon et al., 2019), linking back adhesion and metabolism. Phase 2 clinical data suggests that 17-AAG (Tanespimycin) at a dosage of 340 mg/m^2^ in combination with bortezomib serves as a promising therapeutic option for patients with refractory multiple myeloma (Richardson et al., 2010). Regulation of metabolic components in AML was also studied by implementing dietary restriction coupled with the AMPK inhibitor Compound C, enhancing overall survival reducing the percentage of circulating AML cells *in vivo* (Saito et al., 2015). In addition, metformin administration stimulates the LKB1/AMPK cascade leading to mTORC declination in AML supported by shrinkage in tumor burden (Sujobert et al., 2015). Recent research suggests that resistance to chemotherapy in AML is mediated by metabolic alterations that are short-lived and sufficient to drive patients and patient-derived xenograft models into relapse (Van Gastel et al., 2020). Upon chemotherapeutic intervention specialized leukemic cells prevailed within the bone marrow. The metabolic signature of these cells comprised elevated levels of glutamine, further supporting pyrimidine synthesis. Pyrimidine synthesis, in turn, required a sustained aspartate supply from BM stromal cells. Blocking pyrimidines using Brequinar in combination with lower-dose chemotherapy cleared AML cells from the BM in PDX models (Van Gastel et al., 2020).

Multiple therapeutic options within the BM microenvironment have thus far targeted soluble factors and cell adhesion molecules. The CXCR4-CXCL12 axis was targeted using several individual inhibitors (Burger et al., 2005; Parameswaran et al., 2011; Uy et al., 2017); however, combination therapies proved more effective. For instance, the AML specific peptide E5 (Li et al., 2015) hindered the adhesion and migration of AML cells to the BM by interfering with the CXCR4-CXCL12 pathway. In combination with the chemotherapeutic drugs vincristine and cyclophosphamide, the leukemic burden in mice was reduced, and survival was prolonged (Li et al., 2015). Co-culture of leukemic cells with BM stromal cells confers resistance to the CD44 antibody A3D8 *via* the PI3K-Akt pathway (Chen et al., 2015). Administration of the PI3K-Akt inhibitor, LY294002, along with A3D8, partially resensitizes the AML cells (Chen et al., 2015). α4β1 inhibition reduces chemoresistance of AML and MM cell lines (Rashidi and Dipersio, 2016) and restores CD3-mediated cytotoxicity within the AML BM microenvironment (Nair-Gupta et al., 2020).

## Conclusion

The importance of cell adhesion molecules within the BM is well-acclaimed and continuously advancing as the expression of certain molecules strongly correlates to malignancy. While our understanding of the crosstalk between integrins and these metabolites is improving, there remains a gap in its therapeutic application. Investigating the extent of chemo-sensitization within the BM upon combining adhesion molecules and metabolic inhibitors might bring novel insights into potential therapeutic alternatives.
